# Health Tract, No. 212

**Published:** 1864-09

**Authors:** 


HEALTH TRACT, NO. 212.

### FACTS ABOUT EATING.

If too much food is taken, the stomach can not convert it into a perfect blood material, hence no perfect blood is made, and that being mixed with the other blood in the body makes the whole mass of blood impure; hence, after an over-hearty meal a person "feels bad all over." If the over-eating is habitual, there is always some uncomfortable symptom complained of. Such persons are never well, and although they may eat heartily, they do not get strong nor fill up in flesh; it is because the stomach has been over-taxed and has not the power to extract the nourishment from the food.

When persons do not get strong, although they eat a great deal, they will get stronger by eating one half less; as a sickly servant in attempting to do a large amount of work, does none of it well, whereas, if the task were a light one, the whole of it would have been thoroughly done.

When any uncomfortable feeling is experienced after eating, it is because some article does not "agree with the stomach," that is, can not be digested by it. This always arises from quality or quantity, generally the latter. In such cases take less and less until no discomfort is produced; if no special change is observed, it is because the quality is unsuited to the condition of the stomach, or the general system does not require it.

An article may not agree with the stomach to-day, but may agree with it very well in a few days, weeks, or months afterward, because its distinctive elements may then be needed in the system. Most persons instinctively turn away from roast pork in midsummer—it would make them sick—but in winter time, when the thermometer is near zero, large quantities are eaten with a relish, and no specific discomfort follows. As a general rule, instinct is the best guide, and that which is most relished is the thing which should be eaten; but if some discomfort invariably follows, it should be omitted, at least, until a change of air, season, or occupation.

It is a physical and moral wrong to take a single mouthful, when really it is not wanted; the motive being merely to "eat even," to eat it out of the way, or feeling that if it is not eaten it will be thrown away by the cook. If thus thrown away, some worm, or insect, or animal may get it; if eaten by yourself, it only oppresses the system that much.

The finer food is divided or cut up before swallowed, the sooner, the easier and more perfectly is it digested, for like ice, it is dissolved from without, inwards, and the smaller the pieces the sooner are they melted.

"Bread and butter" and milk are the only two articles of food which have all the elements of nutrition; hence from childhood to extreme old age we are never tired of them.

